# Comparison of the effectiveness of polyethylene glycol with and without electrolytes in constipation: a systematic review and network meta-analysis

**DOI:** 10.1186/s12876-016-0457-9

**Published:** 2016-03-31

**Authors:** Peter Katelaris, Vasi Naganathan, Ken Liu, George Krassas, John Gullotta

**Affiliations:** Gastroenterology Department, Concord Hospital, The University of Sydney, Hospital Rd, Concord, NSW Australia; Centre for Education and Research on Ageing and The Ageing and Alzheimers Institute, University of Sydney and Concord Hospital, Hospital Rd, Concord, NSW Australia; AW Morrow Gastroenterology and Liver Centre, Royal Prince Alfred Hospital, Missenden Road, Camperdown, NSW Australia; Scius Healthcare Solutions Pty Ltd, PO Box 84, Northbridge, NSW Australia; Matraville Medical Centre, 165a Perry St, Matraville, NSW 2036 Australia

**Keywords:** Systematic review, Meta-analysis, Constipation, Polyethylene glycol, Macrogol

## Abstract

**Background:**

Polyethylene glycol is commonly used to manage constipation and is available with or without electrolytes. The addition of electrolytes dates back to its initial development as lavage solutions in preparation for gastrointestinal interventions. The clinical utility of the addition of electrolytes to polyethylene glycol for the management of constipation is not established.

The objective of this systematic review and network meta-analysis (NMA) was to assess the relative effectiveness of polyethylene glycol with (PEG + E) or without electrolytes (PEG) in the management of functional constipation in adults.

**Methods:**

A systematic review was conducted to identify randomised controlled clinical trials that assessed the use of polyethylene glycol in functional constipation. The primary outcome was the mean number of bowel movements per week.

**Results:**

Nineteen studies were included in the NMA (PEG N = 9, PEG + E N = 8, PEG versus PEG + E N = 2; involving 2247 patients). PEG and PEG + E are both effective, increasing the number of bowel movements per week by 1.8 (95 % Crl 1.0, 2.8) and 1.9 (95 % Crl 0.9, 3.0) respectively versus placebo and by 1.8 (95 % Crl 0.0, 3.5) and 1.9 (95 % Crl 0.2, 3.6) respectively versus lactulose. There was no efficacy difference between PEG + E and PEG (0.1, 95 % Crl −1.1, 1.2) and there were no differences in safety or tolerability.

**Conclusions:**

Polyethylene glycol with and without electrolytes are effective and safe treatments for constipation in adults. The addition of electrolytes to polyethylene glycol does not appear to offer any clinical benefits over polyethylene glycol alone in the management of constipation.

## Background

Constipation is a common gastrointestinal symptom with a reported mean prevalence of 15 to 17 % amongst the general population. The prevalence is as high as 81 % amongst older hospitalised patients and 95 % amongst patients taking opioid analgesics [1, 2]. Constipation may adversely impact quality of life and increase the use of healthcare resources [1]. Together these factors make constipation an important health issue that needs effective and safe treatments.

Polyethylene glycol, a minimally absorbed osmotic laxative, is commonly used to manage constipation in both adults and children. It is a mixture of different sized compounds with an approximate mean molecular weight of either 3350 or 4000 g/mol and is available in formulations with the addition of electrolytes (PEG + E) or without electrolytes (PEG). Polyethylene glycol exerts its laxative action by increasing the water content of stools due to its ability to interact with water molecules [3]. Importantly, its use is not associated with marked shifts in water from the body, as it essentially only binds with water that is orally ingested [4]. As it lacks any electrical charge, it does not influence the movement of other solutes [3]. Polyethylene glycol is biologically inert and is not metabolised by colonic bacteria. Therefore, it is expected to exert its full osmotic effect with fewer side effects (such as bloating and flatulence) than the non-absorbable sugar laxatives, as there is no fermentative production of intestinal gas [4, 5].

Polyethylene glycols were first used in lavage solutions in preparation for gastrointestinal interventions such as colonoscopy or bowel surgery [4]. For this indication, they are given in high doses and are generally administered with electrolytes to reduce the risk of large electrolyte shifts [4, 6]. Later, lower doses were used for the management of constipation. More recent formulations were developed without electrolytes to reduce the sodium load, improve taste and potentially patient acceptance and compliance also [3].

The clinical effectiveness of polyethylene glycols in the management of constipation in adults is well established and confirmed in a recent meta-analysis by Belsey et al. [7]. This analysis demonstrated that polyethylene glycol is more effective than placebo and active comparators such as lactulose in the treatment of non-organic constipation. In this analysis however, all polyethylene glycol formulations were treated as the same and it did not provided any insight regarding the clinical utility of the addition of electrolytes to polyethylene glycol. In some countries PEG + E is more widely used than PEG for the management of constipation [8]. The reasons for this are multifactorial and include the perception that PEG + E is a more effective treatment for constipation and safer in terms of preventing electrolyte imbalance. There however is a lack of evidence to support or refute these perceptions.

The objective of this systematic review and network meta-analysis (NMA) was to assess the relative effectiveness of polyethylene glycol with or without electrolytes in the management of functional constipation in adults. The primary end point was the difference in the mean number of bowel movements per week. Secondary endpoints relate to the relative safety, tolerability and compliance or willingness to continue polyethylene glycol therapy.

## Methods

### Literature review

Text word searches were carried out using MEDLINE, MEDLINE in Progress, EMBASE, and the Cochrane databases covering inception to April 2015. Search terms were (constipation) AND (PEG OR polyethylene OR macrogol OR movicol OR idrolax OR miralax OR transipeg OR forlax OR colyte OR golytely OR isocolan OR nulytely) NOT colonoscopy. Studies were included in the final analysis if they met the following criteria: published randomised controlled trials comparing oral polyethylene glycol with placebo or a comparator laxative in patients with constipation. A diagnosis of constipation could be based on clinical symptoms, a physician’s opinion, or the Rome I, II or III diagnostic criteria. Bibliographies of all identified relevant studies and reviews were used to perform a recursive search. Only studies conducted in adults and published in English, excluding conference proceedings, were included in the analysis. Attempts were made to contact the authors of several studies for additional information about their data, with one successful response received.

### Data extraction

Two reviewers were involved in a four-step approach for data collection. All steps were performed independently. First, titles and abstracts of the identified citations were screened to see if they met the study selection criteria. Full texts of potentially relevant articles were reviewed to assess if they met the selection criteria. For the studies that did meet the selection criteria, one reviewer conducted extraction of data using a standardised Excel spreadsheet. A second reviewer independently confirmed the accuracy of the extracted data. As the final step, both reviewers determined if the study was to be included in the systematic review and network meta-analysis. Disagreements were resolved by consensus involving an additional two reviewers.

The following study characteristics were extracted: author; title; journal; publication year; population (adult/paediatric); study design; patient age; characteristics; definition of constipation; inclusion/exclusion criteria; intent to treat population (ITT); per protocol population for defecation frequency; mean duration of constipation prior to study intervention; comparability of study groups; sub-analysis based on age; study duration; study medications, dose and duration of treatment. To avoid comparisons of different subjective composite measures of efficacy, a single objective outcome was selected for the primary analysis – mean number of bowel movements per week. Assessment of defaecation frequency was made after a 2-week treatment period (if available) or at end of treatment (mean plus standard deviation [SD] or standard error [SE]). When data was available for both bowel movements and complete spontaneous bowel movements per week the later data was used in the analysis. Where data was only available graphically, estimates of the values were extracted by scaled measurement. Where means were not available, medians and interquartile ranges were collected. All data were adjusted to mean number of bowel movements per week (plus standard deviation) to allow meaningful comparison between the studies. To assess secondary endpoints, data regarding safety, tolerability, and compliance or willingness to continue therapy were collected.

All included studies were assessed for the risk of bias by two reviewers according to recommendations outlined in the Cochrane Handbook for Systematic Reviews of Interventions [9]. Each potential source of bias was graded as high, low or unclear, relating to whether the potential for bias was low or high. Studies were considered as high quality if all of the criteria were graded as low risk of bias.

### Data synthesis (statistical analysis)

Studies included in the analysis were grouped according to whether the polyethylene glycol investigated included electrolytes (PEG + E) or not (PEG) and then according to the comparator (placebo or different active controls).

For all pairings where there was more than one study a direct estimate of the difference in mean number of bowel movements per week has been obtained using both a Bayesian fixed effects and a Bayesian random effects model (i.e. a standard meta-analysis) using SAS v9.3. In addition, all the available data have been combined using a network meta-analysis with a Bayesian random effects model fitted to assess the relative effectiveness of PEG and PEG + E [10]. NMA was used as it allows an estimation of comparative effects to be made between two treatments that have not been sufficiently investigated by head to head randomised clinical trials [11]. The percentage of simulations where active treatment had a mean stool frequency greater than for control was obtained and is reported as a percentage, ‘probability best’. In this analysis, if the two treatments are equivalent, then the active treatment would be greater than the control 50 % of the time, and hence the ‘probability best’ equals 50 %. If the active is better than the control in all simulations the probability best equals 100 %.

The mean stool frequency was treated as a continuous outcome and hence a generalized linear model with identity link and a normal likelihood distribution was fitted using SAS v9.3 PROC MCMC. Vague (flat) priors were used for all calculations. A normal distribution (0, 106) was used for treatment effects and a uniform (0.01, 5) for inter-study standard deviation. Each analysis was run with 200,000 simulations, 1000 burn-in and thin = 20. The sensitivity of the results to study quality was explored by re-fitting the NMA having excluded all studies with a high risk of bias.

## Results

### Literature search

**Fig. 1:**
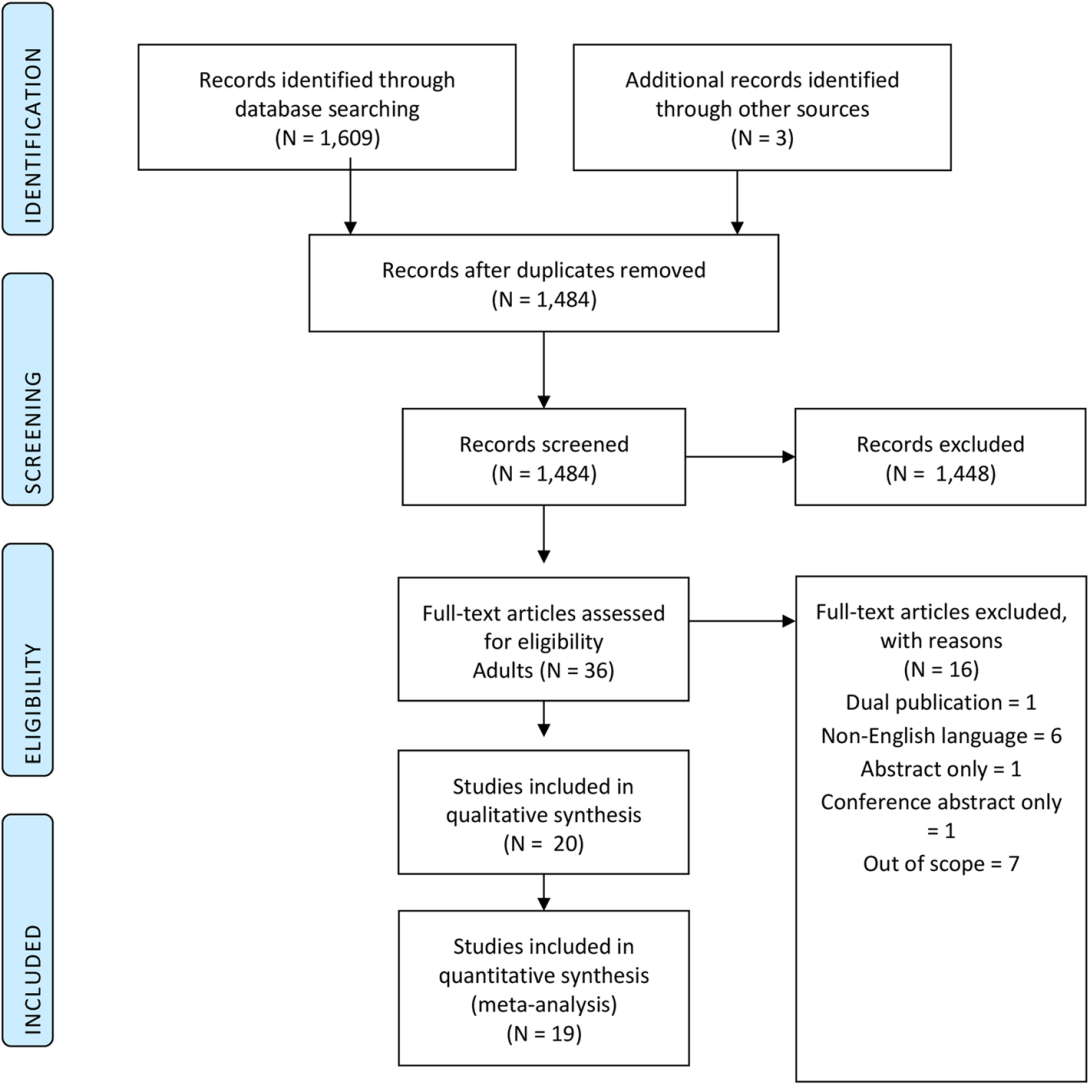
Flow chart of study selection

An overview of the study selection process is summarised in Fig. 1. Literature searches identified 1612 potentially relevant abstracts that after elimination of duplicates was reduced to 1484. After review of the abstracts, 36 full-text publications were assessed of which 20 studies were included in the final systematic review and 19 in the NMA [12–31].

### Study characteristics

Of the 20 qualifying studies; nine studies compared PEG versus placebo (*N* = 7), lactulose (*N* = 1) or serotonin agonist (*N* = 1); nine studies compared PEG + E versus placebo (*N* = 5), lactulose (*N* = 1), serotonin agonist (*N* = 1) or bulk forming laxative (*N* = 1), plus one study involved both an active (lactulose) and placebo control group; and two studies made direct comparisons between PEG and PEG + E. Table 1 summarises the design of each of the individual studies. One study, Corazziaria 2000 [21] was not included in the NMA as all patients had responded to treatment during the run-in phase and had normal bowel function at the time of randomisation. This study however was included in the systematic review to evaluate relevant safety and tolerability data.

**Table 1:** Summary of the included studies

Study	Total (N)	Length of study	Constipation type	Baseline stool frequency (SD)	Concomitant laxative use	Age mean (SD) years	Gender (% male)	Polyethylene glycol formulation and daily dose	Polyethylene glycol (N)	Comparator and daily dose	Comparator (N)
Andorsky RI 1990 [12]	37	2 × 5 days	Chronic	87.5 % ≤ 2 stools/week	Continued use of fibre and bulk-forming agents. Laxatives or enemas for treatment failure: P = 12.5 %, C = 18.8 %	P1: 62	P1: 25 %	PEG3350 + E	High dose 16	Placebo	16
						P2: 58	P2: 19 %	High dose 16 oz	Low dose 16		
								Low dose 8 oz			
Attar A 1999 [13]	115	4 weeks	Chronic	Not specified	Suppositories, micro-enemas allowed. Any use: *P* = 16 %, C = 34 %	P: 55 (24)	P: 15 %	PEG3350 + E	60	Lactulose	55
						C: 55 (22)	C: 22 %	13–39 g		10–30 g	
Awad RA 2010 [14]	47	30 days	Irritable bowel syndrome (IBS-C)	P: 1.3 (0.6)	Not allowed. P: 1 use of glycerine suppository	P: 30.7 (8)	12 %	PEG3350	23	Placebo	24
				C: 1.5 (0.7)	C: 1 use of enema	C: 36.5 (10)		10.35 g			
Bouhnik Y 2004 [15]	65	28 days	Chronic, idiopathic	85 % < 3 stools/week	Suppositories, enemas allowed during washout, stopped 48 hours before baseline. 9 % of patients took concomitant treatment at study entry and all were stopped	57 (18)	14 %	PEG4000 10–30 g	32	Lactulose 10–30 g	33
Chapman RW 2013 [16]	139	28 days	Irritable bowel syndrome (IBS-C)	P: 1.28 (0.912)	Rescue medication bisacodyl if no bowel movement for 3 consecutive days. Mean No. weekly rescue doses: P = 0.30, C = 0.44	41.3 (14.8)	17 %	PEG3350 + E	68	Placebo	71
				C: 1.37 (0.849)				13.8–41.4 g			
Chaussade S 2003 [17]	266	2 weeks	Chronic, idiopathic	P(H): 2.0 (0.9)	Rescue medication suppository if no bowel movement for 3 consecutive days. Stools post-suppository use were not included in study results	52.2 (18.5)	15 %	PEG3350 + E	High dose 69	PEG4000 High dose 20 g	High dose 67
				P(L): 2.2 (1.3)				High dose 11.8 g	Low dose 69	Low dose 10 g	Low dose 65
								Low dose 5.9 g			
				C(H): 2.4 (1.7)							
				C(L): 1.8 (1.0)							
Cinca R 2013 [18]	240	2 weeks	Chronic	P: 0.7 (0.8)	Rescue medication ducosate micro-enema if no bowel movement for 3 consecutive days. Use of micro-enema: P = 0.8 %, C = 3.4 %	P: 40.0 (14.5)	0 %	PEG335 0 + E	120	Prucalopride 1–2 mg (serotonin [5-HT_4_] agonist)	116
				C: 0.7 (0.8)		C: 40.5 (13.2)		13.71–27.42 g			
Cleveland MV 2001 [19]	23	2 × 2 weeks	History of constipation	2.6 (1.75)	Not allowed	47.7	4 %	PEG3350 10.35 g	23	Placebo	23
Corazziari E 1996 [20]	55	4 weeks	Chronic	P: 2.2 (0.5)	Rescue medication, laxatives if no bowel movement for 5 consecutive days. Use of laxatives: P = 16 %, C = 48 %	41.8 (14.8)	P: 32 %	PEG4000 + E 17.5 g	25	Placebo	23
				C: 1.9 (0.8)			C: 13 %				
Corazziari E 2000 [21]	70	20 weeks	Chronic	P: 1.53 (1.35)	Rescue medication, laxatives if no bowel movement for 5 consecutive days. Use of laxatives was less frequent in PEG + E than placebo (P < 0.001)	43 (15)	17 %	PEG4000 + E 35 g	33	Placebo	37
				C: 1.29 (1.04)							
Di Palma JA 1999 [22]	AM: 50 LT 35	10 days	Reported constipation	Not specified	Not allowed	AM: 36.2	AM: 6 %	PEG3350	AM high dose: 50	Placebo	AM: 50
						LT: 75.7	LT: 46 %	AM high dose: 34 g	AM low dose: 50		LT: 17
								AM low dose: 17 g	LT high dose: 17		
								LT high dose: 12 g	LT low dose: 17		
								LT low dose: 6 g			
Di Palma JA 2000 [23]	151	14 days	History of constipation	Not specified	Not allowed	45.2	13 %	PEG3350 17 g	80	Placebo	71
Di Palma JA 2007A [24]	100	4 Weeks	Secondary to medications	Not specified	Fibre or other laxatives not allowed	58	26 %	PEG3350 17 g	46	Placebo	46
Di Palma JA 2007B [25]	304	6 months	Chronic	P: 86.5 % < 3 stools/week	Fibre not allowed. Rescue medication bisacodyl if no bowel movement for 4 consecutive days. Mean use of bisacodyl 5 mg: *P* = 2.8, C = 3.9 tablets/week	53	15 %	PEG3350 17 g	202	Placebo	100
				C: 94.4 % < 3 stools/week							
Di Palma JA 2007C [26]	237	28 days	Chronic	100 % < 3 stools/week	Fibre not allowed. Rescue medication bisacodyl if no bowel movement for 4 consecutive days. Mean use of bisacodyl 5 mg: *P* = 1.4, C = 1.0 tablets/week	46	10 %	PEG3350 17 g	118	Tegaserod 12 mg (serotonin [5-HT_4_] agonist)	116
Freedman MD 1997 [27]	57	3 × 2 weeks	Opioid-induced	Not specified	Additional milk of magnesia or bisacodyl allowed. No difference in use between PEG + E and lactulose	Range 18–50	Not specified	PEG3350 + E 14 g	57	Placebo Lactulose 30 mL	Placebo 57
											Lactulose 57
Klauser AG 1995 [28]	8	2 × 6 weeks	Chronic	Median 3	Sodium picosulfate was allowed except for the last week of each study period. Median drops per day: *P* = 0, C = 4	46 (4)	0 %	PEG4000 60 g	8	Placebo	8
Seinela L 2009 [29]	65	4 weeks	Chronic functional	P: 9.3	Continued use of *Plantago ovata* seeds was allowed. Rescue medication bisacodyl 10 mg suppository if no bowel movement for 3 consecutive days. Use of suppositories: PEG + E = 12.5 %	86	34 %	PEG4000 + E 6–24 g	32	PEG4000 6–24 g	30
				C: 8.4							
					PEG = 3.3 %						
Wang H 2005 [30]	126	2 weeks	Chronic functional	P: 1.18 (0.77)	Not allowed	P: 51. 2 (14.8)	40 %	PEG3350 + E 27.6 g	63	Isphagula husk 7 g (bulk forming)	63
				C: 1.33 (0.68)		C: 50.0 (17.1)					
Zangaglia R 2007 [31]	57	8 weeks	History of constipation amongst Parkinson’s Disease	P: 1.9 (0.56)	Rescue medication, rectal laxatives. Use of rectal laxatives: *P* = 4.3 % week 4 & 0 % week 8	71.0 (6.5)	60 %	PEG4000 + E 7.3–21.9 g	29	Placebo	28
				C: 2.0 (0.6)	C = 9.5 % week 4 & 12.5 % week 8						

*P* Polyethylene glycol group, *C* Comparator group, *AM* Ambulatory healthy outpatients, *LT* Long term, *H* High dose, *L* Low dose

Overall, of the 19 studies included in the NMA, 2247 patients had been randomised to either one of the polyethylene glycols, placebo or active control. All studies had comparable study populations at baseline or used a crossover design. All studies were randomised clinical trials and all but 4 studies were blinded. Four of the studies were assessed as being of low risk of bias based on the recommendations outlined in the Cochrane Handbook for Systematic Reviews of Interventions [9]. (Table 2).

**Table 2:** Individual study results included in the network meta-analysis

Study	Assessment time^a^	Stools per week (number of patients)						Low risk of bias
		PEG + E	PEG	Placebo	Lactulose	Serotonin agonist	Bulk forming	
Awad RA 2010 [14]	30 days		4.1 (*N* = 23)	4.0 (*N* = 24)				Yes
Cleveland MV 2001 [19]	14 days		7.0 (*N* = 23)	3.6 (*N* = 23)				No
Di Palma JA 1999 [22] (AM high dose)	10 days		5.6 (*N* = 50)	3.2 (*N* = 50)				No
Di Palma JA 1999 [22] (AM low dose)	10 days		3.8 (*N* = 50)	3.2 (*N* = 50)				No
Di Palma JA 1999 [22] (LT high dose)	10 days		4.9 (*N* = 17)	4.1 (*N* = 17)				No
Di Palma JA 1999 [22] (LT low dose)	10 days		3.2 (*N* = 17)	4.1 (*N* = 17)				No
Di Palma JA 2000 [23]	14 days		4.5 (*N* = 80)	2.7 (*N* = 71)				No
Di Palma JA 2007A [24]	14 days		8.9 (*N* = 46)	5.6 (*N* = 46)				Yes
Di Palma JA 2007B [25]	14 days		7.9 (*N* = 202)	5.6 (*N* = 100)				No
Klauser AG 1995 [28]	6 weeks		11.0 (*N* = 8)	3.0 (*N* = 8)				No
Andorsky RI 1990 [12] (High dose)	14 days	13.4 (*N* = 16)		7.5 (*N* = 16)				No
Andorsky RI 1990 [12] (Low dose)	14 days	8.1 (*N* = 16)		6.1 (*N* = 16)				No
Chapman RW 2013 [16]	4 weeks	4.4 (*N* = 68)		3.1 (*N* = 71)				Yes
Corazziari E 1996 [20]	4 weeks	4.8 (*N* = 25)		2.8 (*N* = 23)				No
Freedman MD 1997 [27]	14 days	6.9 (*N* = 57)		6.5 (*N* = 57)	5.8 (*N* = 57)			No
Zangaglia R 2007 [31]	8 weeks	6.6 (N = 29)		3.7 (*N* = 28)				No
Chaussade S 2003 [17] (High dose)	2 weeks	6.6 (*N* = 69)	8.2 (*N* = 67)					No
Chaussade S 2003 [17] (Low dose)	2 weeks	6.9 (*N* = 65)	6.0 (*N* = 65)					No
Seinela L 2009 [29]	2 weeks	8.7 (*N* = 32)	9.5 (*N* = 30)					No
Bouhnik Y 2004 [15]	28 days		8.8 (*N* = 32)		7.8 (*N* = 33)			No
Di Palma JA 2007C [26]	28 days		10.4 (*N* = 118)			8.5 (*N* = 116)		No
Attar A 1999 [13]	4 weeks	9.1 (*N* = 60)			6.3 (*N* = 55)			No
Cinca R 2013 [18]	2 weeks	3.2 (*N* = 120)				2.2 (*N* = 116)		Yes
Wang H 2005 [30]	2 weeks	8.5 (*N* = 63)					5.7 (*N* = 63)	No

^a^Assessment time was 14 days after treatment initiated (if available) or at end of treatment

*AM* Ambulatory healthy outpatients, *LT* Long term

In the majority of the studies (*N* = 11) patients had chronic constipation, [12, 13, 15, 17, 18, 20, 25, 26, 28–30] in four studies patients had a history of constipation, [14, 19, 22, 23] in two studies constipation was secondary to medication use [24, 27] and in two studies constipation was related to underlying disease, Parkinson’s Disease [31] and irritable bowel syndrome [16]. Overall more female patients (86.7 %) were assessed in these studies and the average patient age ranged from 30.7 to 86 years.

### Network meta-analysis

**Fig. 2:**
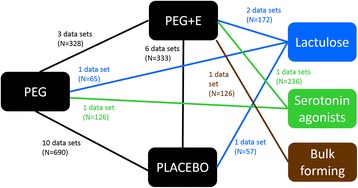
Network formed by interventions and their direct comparisons included in the analyses

Figure 2 presents the network diagram based on the 19 studies included in the NMA, showing a total of 26 connections between the comparators. The individual study results for the mean number of bowel movements per week from the included trials are presented in Table 2.

Some publications contribute more than one data set due to multiple comparator groups, e.g. high and low dose groups, both active and placebo controls, and multiple studies reported within the one publication.

**Fig. 3:**
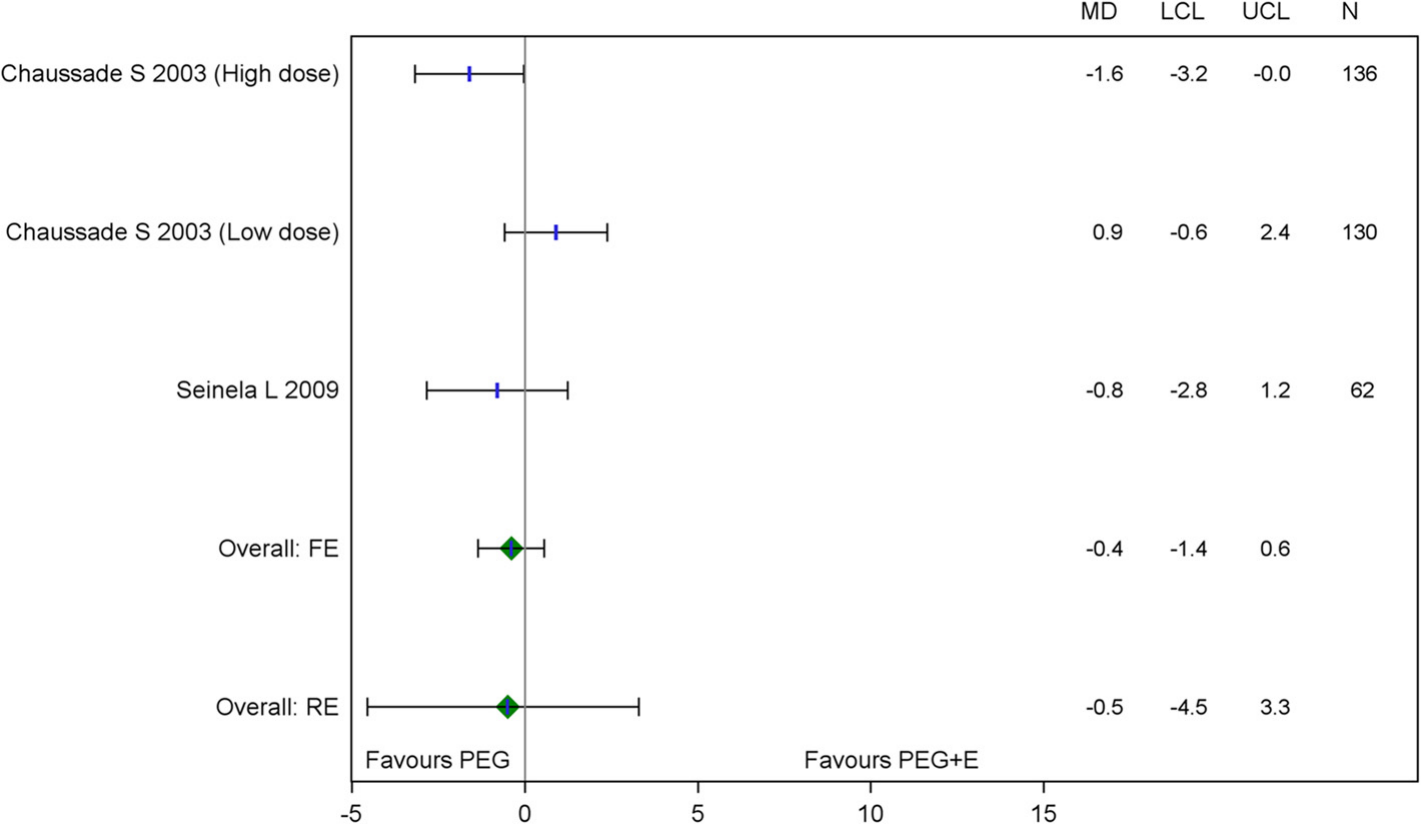
Mean difference in weekly bowel movements PEG + E vs PEG (head-to-head studies)

Table 3 summarises the results for the Bayesian random effects pairwise meta-analysis for direct evidence. The only comparison that was statistically significant was for PEG versus placebo, with PEG increasing the mean number of bowel movements per week by 1.8 (95 % Crl 0.2, 3.6). The direct comparison between PEG and PEG + E failed to show any significant efficacy difference (−0.5, 95 % Crl,-4.5, 3.3) (Fig. 3).

**Table 3:** Results for the direct head-to-head random effects meta-analysis and network meta-analysis

Comparison	Difference in mean stool frequency (direct)	Lower credible limit (direct)	Upper credible limit (direct)	Tau-sq: between study heterogeneity for direct MA	Number of data sets for direct MA	Difference in mean stool frequency (NMA)	Lower credible limit (NMA)	Upper credible limit (NMA)	Probability active treatment is better than comparator in NMA^a^
PEG vs Placebo	1.8	0.2	3.6	2.17	10	1.8	1.0	2.8	100.0 %
PEG + E vs Placebo	2.1	−0.1	4.1	2.09	6	1.9	0.9	3.0	100.0 %
PEG + E vs PEG	−0.5	−4.5	3.3	2.81	3	0.1	−1.1	1.2	58.5 %
PEG vs Lactulose						1.8	0.0	3.5	97.6 %
PEG + E vs Lactulose	1.7	−4.2	4.9	3.45	2	1.9	0.2	3.6	98.6 %
PEG vs Serotonin agonist						1.3	−1.0	3.5	87.7 %
PEG + E vs Serotonin agonist						1.4	−0.9	3.7	89.2 %
PEG vs Bulk forming						2.6	−0.8	5.8	93.6 %
PEG + E vs Bulk forming						2.6	−0.5	5.8	95.1 %

*NMA* network meta-analysis, *MA* meta-analysis ^a^Note a probability of 50 % equates to no difference between the two therapies

Direct head-to-head random effects meta-analysis was performed when there was two or more published studies

**Fig. 4:**
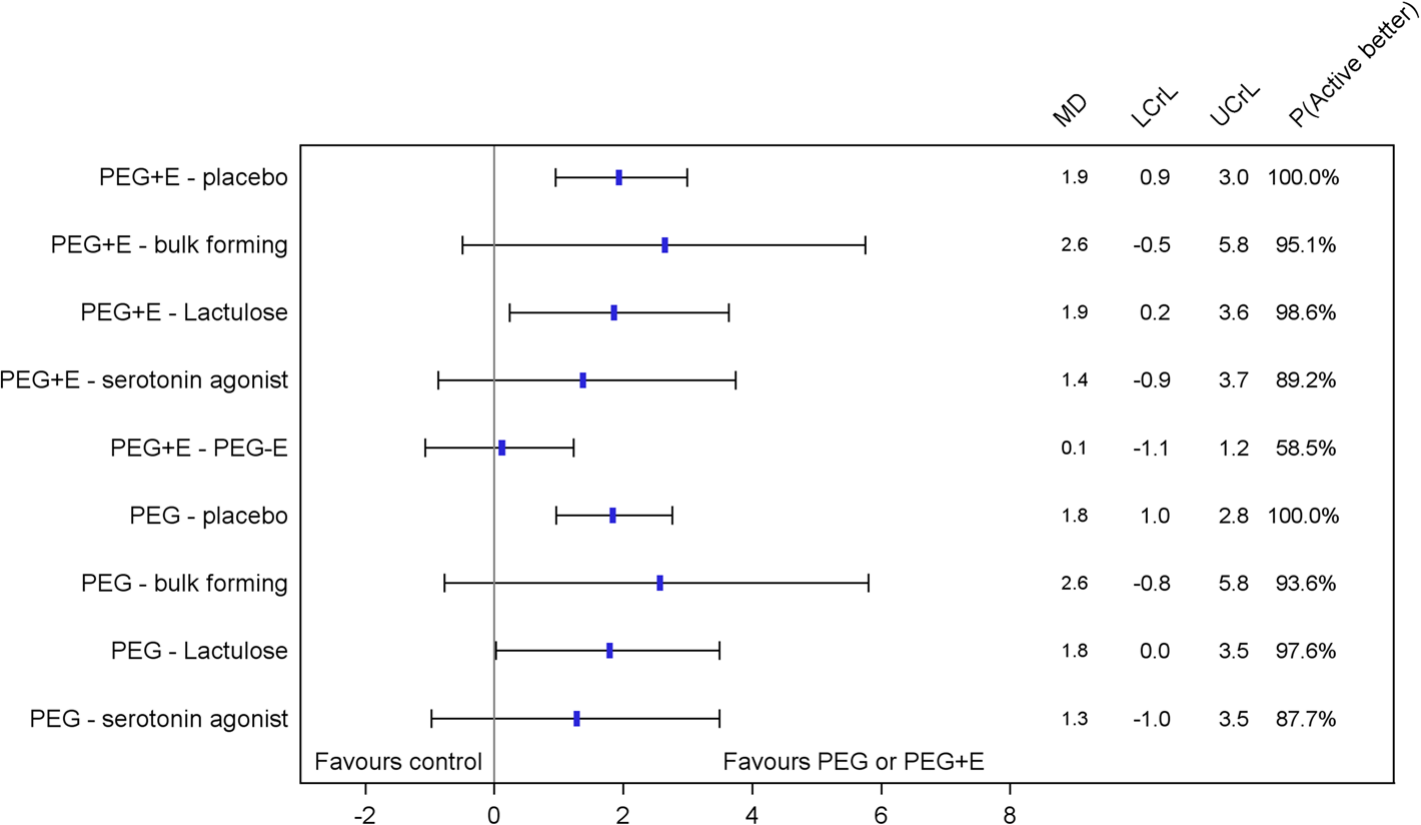
Pairwise comparisons for PEG and PEG + E from the network meta-analysis

Table 3 and Fig. 4 summarise the results of the NMA. With the inclusion of more data in the NMA, this analysis suggests that both PEG and PEG + E are more effective than both placebo and lactulose, increasing the mean number of bowel movements per week by 1.8 (95 % Crl 1.0, 2.8) and 1.9 (95 % Crl 0.9, 3.0) respectively versus placebo and by 1.8 (95 % Crl 0.0, 3.5) and 1.9 (95 % Crl 0.2, 3.6) respectively versus lactulose. Comparisons of PEG and PEG + E with bulk forming laxatives and serotonin (5-HT_4_) agonists were not statistically significant. The direct comparison of PEG + E and PEG suggests that the difference in the mean number of bowel movements per week is negligible and not statistically significant (0.1, 95 % Crl −1.1, 1.2). The relative effectiveness of both PEG and PEG + E versus placebo was maintained with sensitivity analysis including only studies with a low risk of bias.

### Safety and tolerability

Twelve studies included in the systematic review provided data on the safety of polyethylene glycol [13, 17–19, 21–25, 29–31] and all but one study provided tolerability data [12–27, 29–31] (Table 4). Overall, there were no clinically relevant changes in laboratory measures or vital signs, with the exception of one case of mild hypokalaemia in a patient taking PEG + E with concurrent diuretic use [13]. Polyethylene glycol with or without electrolytes was well tolerated with most events being mild to moderate in severity. The more common adverse events included abdominal pain, diarrhoea, loose stools, nausea and abdominal distension. In addition, two PEG + E studies reported issues regarding poor taste as an adverse event, [12, 31] whilst taste was not reported as an issue with the use of PEG. Amongst the placebo-controlled studies, three of the seven PEG [14, 23, 24] and two of the five PEG + E studies [20, 21] specifically reported no differences in tolerability versus placebo, whilst 1 study for PEG [25] and two studies for PEG + E [16, 31] reported a higher incidence of side effects versus placebo. Studies that directly compared PEG with PEG + E demonstrated no differences in tolerability, [17, 29] with the exception that in one study there were four serious events leading to discontinuation with PEG + E, but no cases with PEG [29]. Eleven patients (1.8 %) discontinued therapy due to adverse events with PEG + E compared to three patients (0.4 %) with PEG. Reasons for discontinuation with PEG + E included abdominal pain, abdominal rigidity, abdominal bloating, nausea, diarrhoea, anal fissure and poor taste. Reasons for discontinuation with PEG were abdominal pain and distention.

**Table 4:** Safety and tolerability of polyethylene glycols from the individual studies

Study	Type of polyethylene glycol	Comparator	Safety signals (Laboratory data, vital signs)	Tolerability (Adverse events)
Andorsky RI 1990 [12]	PEG3350 + E	Placebo	NA	Adverse events with PEG + E were infrequent and generally tolerable and included; cramping, gas, nausea, loose stools, and unpleasant taste.
Attar A 1999 [13]	PEG3350 + E	Lactulose	No significant changes in laboratory measurements; except for 1 case of mild hypokalaemia with concurrent diuretics. In the 2 month open label extension study, lower mean serum folate levels, but all values were within the normal range.	No differences in tolerability between the two groups, but flatus was less frequently reported with PEG + E. 2 adverse events leading to PEG + E withdrawal; acute diarrhoea with vomiting and fever; and abdominal pain. Additional 4 adverse events leading to drug withdrawal in the extension study; acute diarrhoea with fever (1), abdominal pain (2); vomiting (1).
Awad RA 2010 [14]	PEG3350	Placebo	NA	No difference in tolerability of PEG vs placebo. 1 case of abdominal pain with PEG.
Bouhnik Y 2004 [15]	PEG4000	Lactulose	NA	No serious adverse events were reported. 3 PEG patients discontinued therapy due to adverse events; abdominal pain or abdominal distension.
Chapman RW 2013 [16]	PEG3350 + E	Placebo	NA	More patients taking PEG 3350 + E experienced adverse events compared to placebo (38.8 % vs 32.9 %). No serious adverse events. The most common drug-related adverse events (>3 %); abdominal pain (4.5 %), diarrhoea (4.5 %). 2 patients discontinued PEG + E due to adverse events; abdominal rigidity (1), flatulence and abdominal pain (1).
Chaussade S 2003 [17]	PEG3350 + E and PEG4000	PEG4000	No clinical issues reported.	No differences in tolerability. Common GI adverse events; dose-related diarrhoea, distention, flatulence, abdominal pain.
Cinca R 2013 [18]	PEG3350 + E	Prucalopride	No clinically significant differences in laboratory measurements, vital signs or ECG.	68.3 % of patients taking PEG + E experienced a treatment-emergent adverse event, mostly mild-moderate intensity. 5.3 % of the events were possibly or probably related to PEG + E. Events included; headache (36.7 %), nausea (5.8 %), vomiting (2.5 %) and abdominal pain (2.5 %), UTI (3.3 %). Adverse event were generally more common with prucalopride.
Cleveland MV 2001 [19]	PEG3350	Placebo	No clinically significant differences in blood chemistry, CBC, or urinalysis.	No serious adverse events. Three cases of loose stools or mild diarrhoea with PEG.
Corazziari E 1996 [20]	PEG4000 + E	Placebo	NA	No difference in tolerability of PEG + E vs placebo.
Corazziari E 2000 [21]	PEG4000 + E	Placebo	No significant changes in heart frequency, blood pressure, blood count or laboratory measurements.	No difference in tolerability of PEG + E vs placebo. 2 discontinuations due to adverse events; abdominal bloating and fissura in the anus. Most common adverse events were nausea and epigastric pain/discomfort.
Di Palma JA 1999 [22]	PEG3350	Placebo	No clinically significant changes in laboratory measurements.	Ambulatory care patients: dose-related diarrhoea or loose stools. Long-term care patients: 5 serious adverse events, but all were due to pre-existing conditions and not PEG use.
Di Palma JA 2000 [23]	PEG3350	Placebo	No statistically or clinically significant differences in laboratory measurements.	No difference in tolerability of PEG vs placebo.
Di Palma JA 2007A [24]	PEG3350	Placebo	No clinically significant changes in vital signs, physical examination, weight, or laboratory measurements.	No statistical difference in tolerability of PEG vs placebo.
Di Palma JA 2007B [25]	PEG3350	Placebo	No clinically significant changes in laboratory measurements.	No differences in adverse events between PEG and placebo except for gastrointestinal complaints (PEG 39.7 %, placebo 25 %, *P* = 0.015). GI events included abdominal distension, diarrhoea, loose stools, flatulence, and nausea. Most events were mild or moderate. No difference in tolerability of PEG + E vs placebo amongst elderly patients.
Di Palma JA 2007C [26]	PEG3350	Tegaserod	NA	No serious adverse events. Adverse events (>3 %) with PEG were; GI (30.8 %), diarrhoea (20 %) and nausea (5.2 %).
Freedman MD 1997 [27]	PEG3350 + E	Placebo Lactulose	NA	No difference in frequency of gas or severe cramping with PEG + E vs control.
Klauser AG 1995 [28]	PEG4000	Placebo	NA	NA
Seinela L 2009 [29]	PEG4000 + E and PEG4000	PEG4000	Small, but not clinically relevant changes in plasma sodium level; PEG mean decrease from 138.8 to 137.7 mmol/L; PEG + E mean increase from 138.6 to 138.9 mmol/L (P = 0.012). No other significant differences between the groups in any of the other electrolyte or laboratory safety variables, or in heart rate, blood pressure or weight.	Low incidence of mild to moderate adverse events in both groups. Four serious adverse events with PEG + E; 1 leading to discontinuation of PEG + E, but none with PEG.
Wang H 2005 [30]	PEG3350 + E	Isphagula husk	No change in mean sodium, potassium or chloride ion levels.	No differences in adverse events between PEG + E and isphagula husk. No serious events. Most common adverse event for PEG + E was dizziness (5 %).
Zangaglia R 2007 [31]	PEG4000 + E	Placebo	No clinically significant changes in haematology, serum biochemistry, or urinalysis.	A higher rate of withdrawals with PEG + E vs placebo (31 % vs. 18 %). 4 drug-related discontinuations were due to nausea, diarrhoea, poor treatment compliance due to the taste or volume of preparation.

*NA* No applicable data reported, *AM* Ambulatory healthy outpatients, *LT* Long term

### Compliance, willingness to continue therapy

Only two studies provided data on patient compliance or willingness to continue polyethylene glycol therapy. In a placebo-controlled study, compliance was lower with PEG + E as assessed by the mean number of sachets used per week [16]. In a direct comparison of PEG and PEG + E, more patients were willing to continue with PEG therapy (85 % vs 63 %), but this difference was not statistically significance (*p* = 0.07) [29].

## Discussion

The aim of this network meta-analysis was to assess the relative effectiveness of polyethylene glycol with and without electrolytes in the management of functional constipation. The addition of electrolytes to polyethylene glycol did not enhance clinical effectiveness compared to polyethylene glycol alone.

This NMA and the direct head-to-head random effects meta-analysis are consistent and confirmed that both PEG and PEG + E are effective treatments for constipation. Comparisons with placebo were highly significant, with PEG and PEG + E increasing the number of bowel movements per week by 1.8 and 1.9 respectively. These results are consistent with previously published meta-analysis where polyethylene glycol (PEG and PEG + E grouped together) was found to increase the mean number of bowel movements per week by 1.98 versus placebo [7]. Our results also suggest that both PEG and PEG + E are more effective than lactulose a result that is also consistent with other meta-analyses [7, 32, 33]. The extent of improvement was again similar for both forms of polyethylene glycol.

No safety signals emerged that would suggest that the addition of electrolytes provided any safety benefits to the use of polyethylene glycol in the management of constipation. Although most of the studies in this systematic review were of short duration, this finding is consistent with a long-term open label study of PEG. Over the 12 month study period no clinically significant changes in haematology or blood chemistry, particularly electrolytes, were observed amongst the whole study population or in older participants [34].

Overall the tolerability of PEG and PEG + E was found to be good and often comparable to placebo. The majority of adverse events were gastrointestinal and were rated as mild to moderate in severity. Withdrawals due to adverse events were uncommon with both PEG + E and PEG. From a safety and tolerability perspective the addition of electrolytes to polyethylene glycol does not appear to provide any additional clinical benefits in the management of constipation.

PEG is a tasteless and odourless and can be mixed with the beverage of the patient’s choice [3]. Although the data identified in this systematic review is limited, one double-blind study in 100 adults reported that PEG was rated as significantly better tasting the PEG + E (*p* < 0.0001) with 84 subjects preferring the taste of PEG whilst only seven subjects preferred PEG + E [35]. Palatability may affect willingness to adhere to therapy [33, 35]. In addition, there is some evidence of the absorption of electrolytes from low doses of PEG + E [23] which may need to be considered in patients with restricted sodium diets.

### Limitations

As with any systematic review, the quality of the studies and the heterogeneity of the study populations included in the analysis present a limitation of this study. There were only four studies considered to have a low risk of bias. Five studies were crossover studies, only two which had washout periods, [11, 27] hence there is a risk of a carry-over effect. In one study the median number of bowel movements per week were reported rather than the mean. Attempts to contact the authors for mean data were unsuccessful and the median has been used as the mean since in a normal distribution these two values would be equal. Another potential limitation is that constipation is a subjective complaint and we have used only one objective measure, the difference in mean number of bowel movement per week, to assess the relative efficacy of the different treatments. It was not possible to conduct a network meta-analysis on the secondary end point of compliance due to the limited data and due to differences in the way this was measured where available. The planned age-related subanalysis was not performed due to the lack of data specifically reported amongst elderly patients.

### Implications for future research

Despite the high prevalence of constipation in the elderly and the frequent use of laxatives in this patient population, there is a paucity of clinical trials evaluating the safety and efficacy of polyethylene glycol in the elderly. This represents an important evidence gap.

## Conclusions

This network meta-analysis has confirmed that polyethylene glycol with and without electrolytes are effective and safe treatments for constipation in adults. There was no difference in the number of bowel movements per week and there appears to be no differences with respect to safety or tolerability between the two preparations. The addition of electrolytes in this clinical setting does not appear to offer any clinical benefits over polyethylene glycol alone.

## Abbreviations

ITT: intention to treat
NMA: network meta-analysis
PEG: polyethylene glycol without electrolytes
PEG + E: polyethylene glycol with electrolytes
SD: standard deviation
SE: standard error
